# Registered report: The common feature of leukemia-associated IDH1 and IDH2 mutations is a neomorphic enzyme activity converting alpha-ketoglutarate to 2-hydroxyglutarate

**DOI:** 10.7554/eLife.12626

**Published:** 2016-02-26

**Authors:** Oliver Fiehn, Megan Reed Showalter, Christine E Schaner-Tooley, Reproducibility Project: Cancer Biology, Elizabeth Iorns

**Affiliations:** Science Exchange, Palo Alto, United States; Mendeley, London, United Kingdom; Science Exchange, Palo Alto, United States; Science Exchange, Palo Alto, United States; Center for Open Science, Charlottesville, Virginia; Science Exchange, Palo Alto, United States; Center for Open Science, Charlottesville, Virginia; 1 West Coast Metabolomics Center, University of California, Davis, Davis, United States; 2 University of Louisville School of Medicine, Louisville, United States; Weill Cornell Medical College, United States

**Keywords:** Reproducibility Project: Cancer Biology, methodology, IDH1, IDH2, acute myeloid leukemia, 2-HG, Human

## Abstract

The Reproducibility Project: Cancer Biology seeks to address growing concerns about reproducibility in scientific research by conducting replications of selected experiments from a number of high-profile papers in the field of cancer biology. The papers, which were published between 2010 and 2012, were selected on the basis of citations and Altmetric scores (Errington et al., 2014). This Registered Report describes the proposed replication plan of key experiments from “The common feature of leukemia-associated IDH1 and IDH2 mutations is a neomorphic enzyme activity converting alpha-ketoglutarate to 2-hydroxyglutarate” by Ward and colleagues, published in Cancer Cell in 2010 (Ward et al., 2010). The experiments that will be replicated are those reported in Figures 2, 3 and 5. Ward and colleagues demonstrate the mutations in isocitrate dehydrogenase 2 (*IDH2*), commonly found in acute myeloid leukemia (AML), abrogate the enzyme’s wild-type activity and confer to the mutant neomorphic activity that produces the oncometabolite 2-hydroxyglutarate (2-HG) (Figures 2 and 3). They then show that elevated levels of 2-HG are correlated with mutations in *IDH1* and *IDH2* in AML patient samples (Figure 5). The Reproducibility Project: Cancer Biology is a collaboration between the Center for Open Science and Science Exchange and the results of the replications will be published by *eLife*.

**DOI:** http://dx.doi.org/10.7554/eLife.12626.001

## Introduction

Mutations in the metabolic enzymes isocitrate dehydrogenase 1 (*IDH1*) and *IDH2* genes, which catalyze the production of α-ketoglutarate (α-KG) from isocitrate, have been associated with numerous forms of cancer (Krell et al., 2013) leading to exploration of how changes in their function could be linked to the development of tumors. All known mutations alter key residues in both proteins that decrease the enzyme’s affinity for isocitrate, leading to the theory that the loss of IDH function perturbs the equilibrium of α-KG, negatively affecting various α-KG dependent enzymes (Zhao et al., 2009). However, work from the Thompson group determined that the tumor-associated mutations actually created a neomorphic function; rather than catalyzing the production of α-KG, mutant IDH proteins produce the oncometabolite 2-hydroxyglutarate (2-HG) (Ward et al., 2012). Dang and colleagues first described this neomorphic function and demonstrated a correlation between 2-HG levels and glioma samples harboring *IDH* mutations (Dang et al., 2009). In their 2010 Cancer Cell paper, Ward and colleagues further confirm these findings and extend the association of 2-HG levels and *IDH* mutations to acute myeloid leukemia (AML) (Ward et al., 2010).

In Figure 2, Ward and colleagues transfected 293T cells with either wild type or mutant forms of *IDH2*. They assessed cell lysates for their ability to generate NDPH in the presence of isocitrate (Figure 2A) or to consume NADPH in the presence of α-KG (Figure 2B). Their data indicated that cells transfected with IDH2^WT^ generated NADPH in the presence of isocitrate, and did not consume much NADPH in the presence of α-KG, consistent with its canonical function of converting isocitrate to α-KG. However, IDH2^R172K^ displayed the opposite effect, indicating that it was able to consume NADPH in an α-KG dependent manner. These data were the first suggesting that the mutant form of IDH2 might have a neomorphic function. This key experiment will be replicated in Protocol 1.

In Figure 3, Ward and colleagues use gas-chromatography mass spectrometry (GC-MS) to identify a novel function of IDH2^R172K^. They identified a unique peak in the lysates of cells transfected with IDH2^R172K^ that corresponded to the retention time of the metabolite 2-hydroxyglutarate (2-HG). They confirmed the metabolite identity by mass spectrometry. These data provide evidence that the mutant form of IDH2 leads to 2-HG production. This key experiment will be replicated in Protocol 2.

In Figure 5, Ward and colleagues examined the correlation between AML patient samples carrying *IDH* mutations and the levels of 2-HG found in those samples. They showed that patient samples carrying *IDH* mutations contained higher levels of 2-HG than samples from patients with WT *IDH* genes. This key experiment will be replicated in Protocol 3.

Several groups’ work has supported the results of Ward and colleagues, who themselves confirmed and extended their initial findings in subsequent reports (Ward et al., 2011; 2013). Leonardi and colleagues confirmed that mutant forms of *IDH*, specifically *IDH1*, did not perform the canonical forward reaction converting isocitrate to α-KG (Leonardi et al., 2012). Using magnetic resonance spectroscopy, Izquierdo-Garcia and colleagues confirmed that transfection of cells with mutant *IDH* forms increased the levels of 2-HG (Izquierdo-Garcia et al., 2015), while Jin and colleagues demonstrated similar findings for *IDH1* and *IDH2* mutants (Jin et al., 2011). Evaluating 2-HG levels in astrocytomas and gliomas harboring various *IDH1* mutations, Pusch and colleagues also showed that any mutations in *IDH1* correlated with increased levels of 2-HG in human patient samples (Pusch et al., 2014), a trend also observed by Juratli and colleagues (Juratli et al., 2013).

Discovery of IDH neomorphic function, resulting in the production of the 'oncometabolite' 2-HG, opened many avenues of research into how the production of excess 2-HG could impact tumorigenesis. Figueroa and colleagues expanded upon the foundation laid by Ward and colleagues and determined that excess 2-HG was correlated with changes in global methylation patterns (Figueroa et al., 2010). Xu and colleagues showed that 2-HG was able to competitively inhibit many α-KG dependent enzymes, including several histone demethylases, and that exogenous 1-HG was able to inhibit histone demethylation (Xu et al., 2011). Lu and colleagues also observed this correlation between 2-HG levels and perturbations in global histone methylation patterns, and went on to show that this resulted in impaired cellular differentiation (Lu et al., 2012).

## Materials and methods

Unless otherwise noted, all protocol information was derived from the original paper, references from the original paper, or information obtained directly from the authors.

### Protocol 1: Assessing the α-ketoglutarate dependent NADPH consumption of wild-type or mutant IDH2

In this protocol, 293T cells are transfected with empty vector, IDH2^WT^, or IDH2^R172K^. Lysates are generated from these cells and their ability to produce NADPH from NADP+ and isocitrate is assayed (Figure 2A). The same lysates are also assayed for their ability to consume NADPH in the presence of 0.5 mM α-ketoglutarate (α-KG) (Figure 2B). Expression of the transfected protein will be confirmed by Western blot (Figure 2C).

#### Sampling

Oxidative and reductive activity (Figures 2A and B):

- Experiment has three conditions. Each will be performed with seven biological replicates and three technical replicates of each condition at each time point for a final power of at least 80%.
    - Condition 1: 293T cells expressing *IDH2^WT^*
    - Condition 2: 293T cells expressing *IDH2^R172K^*
    - Condition 3: 293T cells expressing empty pCDNA3 vector
  o Each lysate will be assessed for cell’s ability to reduce NADP^+^ and oxidate NADPH
  o See Power Calculations section for details.

Confirmatory Western Blot (Figure 2C)

- This is a quality control experiment and is not being powered to detect a specific effect size. Western blots will be performed alongside each biological replicate.
- Western blotting of each lysate will be performed for the following proteins
  - IDH2
  - IDH1
  - Actin [additional]

#### Materials and reagents

**Table:** 

Reagent	Type	Manufacturer	Catalog #	Comments
293T cells	Cells	ATCC	CRL-3216	Original source unspecified
Dulbecco’s modified Eagle’s medium (DMEM)	Media	Invitrogen	11965118	Original unspecified
FBS	Reagent	Hyclone	SH30071.03	Replaces FBS from CellGro
IDH2^WT^ ORF in pCMV6	Plasmid	Origene	RC201152	
IDH2^R172K^ ORF in pCMV6	Plasmid	Origene	RC400103	
pCDNA3	Plasmid	Invitrogen	V790-20	
Lipofectamine 2000	Reagent	Invitrogen	11668027	
M-Per Mammalian protein extraction reagent	Reagent	Pierce	78503	
Aprotinin	Reagent	Sigma	248614	Original protease inhibitor cocktail unspecified
AEBSF	Reagent	EMD Millipore	101500-100MG	
Leupeptin	Reagent	Sigma	L2884-100mg	
Pepstatin A	Reagent	EMD Millipore	516481-100MG	
NaOV	Reagent	Sigma	450243-50G	Original unspecified
NaF	Reagent	Sigma	215309-50G	
Sonicator	Equipment	VCR	75HT	Original unspecified
Refrigerated microcentrifuge	Equipment	Labnet International, Inc	PrismR	Original unspecified
Tris-HCl	Reagent	BioRad	BR0011	Original unspecified
MnCl2	Reagent	M87-100	Fisher	Original unspecified
EDTA	Reagent	VWR	EM-4050	Original unspecified
ß-NADP+	Reagent	MP Biomedicals	ICN10116680	Original unspecified
ß-NADPH	Reagent	Sigma	10107824001	Original unspecified
D-(+)-threo-isocitrate	Reagent	Sigma	I1252	
Spectrophotometer	Instrument	Molecular Devices	Filter Max F5 Multi-mode Microplate Reader	Original unspecified
6-well tissue culture plates	Materials	E& K Scientific	27160	Original unspecified
96 well plates	Materials	Fisher (Costar)	07-200-656	Original unspecified
Tric-HCl	Reagent	BioRad	BR0011	Original unspecified
Glycerol	Reagent	VWR	EM-4760	Original unspecified
ß-mercaptoethanol	Reagent	Sigma	M6250-250mL	Original unspecified
Sodium dodecyl sulfate (SDS)	Reagent	Sigma	L3771-100G	Original unspecified
Bromophenol blue	Reagent	Sigma	B0126-25G	Original unspecified
Protogel	Reagent	Fisher/National Diagnostics	50-899-90119	Original unspecified
APS	Reagent	Sigma	248614	Original unspecified
TEMED	Reagent	Fisher	BP150-100	Original unspecified
nitrocellulose	Materials	BioRad	162-0112	Original unspecified
Anti-IDH2 antibody (mouse monoclonal)	Primary Antibody	Abcam	ab55271	
Anti-IDH1 antibody (goat polyclonal)	Primary Antibody	Santa Cruz	sc49996	
Anti-Actin antibody (rabbit monoclonal)- HRP conjugated	Primary Antibody	Cell Signaling	12620	Not included in original.
ECL Mouse IgG, HRP-linked whole Ab (from sheep)	Secondary Antibody	GE Healthcare	NA931V	
HRP conjugated rabbit anti-goat antibody	Secondary Antibody	Invitrogen	811620	Original unspecified
Protein ladders	Reagent	Cell Signaling Tech.	7727L	Original unspecified
		Gold Biotech	p007-1500	Original unspecified
ECL reagent	Reagent	Fisher Scientific	PI34096	Original unspecified
Endo-free maxiprep kit	Reagent	Qiagen	12362	Original unspecified
α-ketoglutarate	Reagent	Sigma	75892-25G	Original unspecified
DC Protein Assay Kit	Kit	BioRad	5000112	Original unspecified
Alpha innotech imager	Equipment	Alpha Innotech	Alphaimager 2200	
sodium azide	Reagent	Sigma	S2002-5G	Original Unspecified
Ponceau stain	Reagent	Quality Biological	50-751-6798	Additional reagent

#### Procedure

##### Notes

- 293T cells are grown in DMEM supplemented with 10% FBS at 37°C in 5% CO_2_
- Cells will be sent for STR profiling and mycoplasma testing.

1. Confirm insert identity by sequencing.
  - Origene clones are shipped with two sequencing primers.
2. Sub-clone IDH2^WT^ and IDH2^R172K^ from the Origene pCMV6-Entry vectors into pcDNA3.
  - Confirm insert identity by sequencing.
  - Confirm vector integrity by agarose gel electrophoresis.
3. Grow up and use an endo-free maxiprep kit to prep the following vectors:
  - pcDNA3
  - pcDNA3-IDH2^WT^
  - pcDNA3-IDH2^R172K^
4. Seed 0.25-1x10^6^ 293T cells per well of a 6-well plate in growth medium without antibiotics.
  - Grow overnight.
  - Confirm cells at 70–80% confluency by light microscopy at time of transfection.
5. Transfect 293T cells with pcDNA3, pcDNA3-IDH2^WT^, pcDNA3-IDH2^R172K^ with Lipofectamine 2000 according to manufacturer instructions for a 6-well plate.
  - As per manufacture’s instructions 1 µg plasmid DNA per well in a 6-well plate for 70–80% confluent 293T cells.
  - Transfect 1 well (or plate if reaction needs to be scaled up) for each construct
    - This will be one biological replicate
6. 48 hr after transfection, remove medium from cells, wash with PBS, and lyse in 1 ml/well of mammalian protein extraction reagent containing protease inhibitor cocktail (aprotinin, AEBSF, leupeptin and pepstatin A, all at 1:1000) and phosphatase inhibitor cocktails (NaOV, Pepstatin A, Leupeptin, AEBSF, NaF, aprotinin) at 4°C or on ice.
7. Collect lysate and sonicate.
  - Perform test for optimal conditions as follows.
    - Sonication for 5 min
    - Sonication for 10 min
  - Centrifuge lysate in refrigerated microcentrifuge at 14000x*g* at 4°C for 10 min.
  - Collect supernatants and measure the protein concentration of each using the DC Protein Assay Kit II according to the manufacturer’s instructions.
  - Will need >50 µg total protein to proceed
    - If 50 µg total protein is not achieved the reaction will be scaled to a 25 cm plate. These conditions will be used for the subsequent replicates without any further optimization.
    - If further optimization is needed, the experiment will not proceed to step 7 until this is achieved.
  - Aliquot lysate protein for measuring IDH oxidative (Step 9) and reductive activity (step 10) and for examining expression of IDH2^WT^, IDH2^R172K^ by western blot (step 11).
8. Measuring IDH oxidative activity:
  - Mix 0.3 µg of each protein lysate with 200 µl of assay buffer solution in a 96-well plate. Each condition should be plated in triplicate.
    - Assay buffer solution: 100 mM Tris-HCl buffer (pH 7.5), 1.3 mM MnCl_2_, 0.33 mM EDTA, 0.1 mM ß-NADP^+^, 0.1 mM D-(+)-*threo*-isocitrate
    - Include buffer lacking lysate protein to determine background reading.
  - Put mixtures in spectrometer and measure absorbance at 340 nm every 20 s for 30 min.
  - Use absorbance readings at 5 min intervals for analysis.
    - An exploratory investigation of all data will be used in the analysis as well.
9. Measuring IDH reductive activity:
  - Mix 3 µg of each protein lysate with 200 µl of assay buffer solution in a 96-well plate. Each condition should be plated in triplicate.
    - Assay buffer solution: 100 mM Tris-HCl buffer (ph 7.5), 1.3 mM MnCl_2_, 0.01 mM ß-NADPH, 0.5 mM α-ketoglutarate
    - Include buffer lacking lysate protein to determine background reading.
  - Put mixtures in spectrometer and measure absorbance at 340 nm every 20 min for 3 hr.
10. Western blot to confirm protein expression:
  - Add sample buffer and boil lysates to prepare for loading.
    - Sample buffer: 0.5 mL 1 M TrisCl, pH 6.8, 1 mL glycerol, 0.5 mL ß-mercaptoethanol, 0.24 g SDS, 0.1 mL 1% bromophenol blue.
    - Add 30 µg of protein per well by diluting protein to same concentrations (based on protein quantification results) in 10 µL of lyse buffer and added 20 µL of sample buffer
    - Place at 65˚C for 15 min.
  - Separate 20–30 µg of protein per lane on an 8% SDS-PAGE gel with protein ladder.
    - Run through the stacker at 45 mAmp/gel, then increase to 300 V for 3 hr.
  - Transfer to nitrocellulose membrane.
    - Transfer at 100 A for 1 hr 40 min in 2.5 mM Tris, 19 mM glycine in 20% methanol.
    - Wash membrane in deionized water then wash in 1X TBST.
    - Confirm protein transfer with Ponceau stain.
  - Block membrane with 5% milk/0.2% azide in TBST for 30 min at room temperature.
  - Incubate with the following primary antibodies using the manufacturer’s recommended dilution. Following antibodies will be probed at one time
    - Mouse anti-IDH2; 37 kDa
    - Goat anti-IDH1; 47 kDa
  - Incubate with appropriate secondary antibodies using manufacture’s recommended dilutions
    - HRP-conjugated sheep anti-mouse
    - HRP-conjugated rabbit anti-goat
      1. The anti-actin antibody is HRP conjugated and a secondary antibody incubation is not necessary.
  - Treat membranes with ECL reagent according to manufacturer’s recommendations and image.
  - Between antibody incubations, inactivate HRP activity by incubating with a final concentration of 1mM sodium azide in blocking buffer.
    - Shake at room temp for 1 hr.
    - Wash membrane 3 x 5 min in 1X TBST.
    - Incubate with ECL reagent as directed by the manufacturer and image at a time point of at least 5 min to confirm HRP inactivation
    - Save blank image
  - Incubate with Rabbit anti-actin-HRP; 45 kDa [additional] to evaluate loading control
  - Treat membranes with ECL reagent according to manufacturer’s recommendations and image.
11. Repeat steps 6–9 independently six additional times.

#### Deliverables

- Data to be collected:
  - Sequencing reads and agarose gel images confirming vector identity and integrity
    - pcDNA3
    - pcDNA3-IDH2^WT^
    - pcDNA3-IDH2^R172K^
  - Raw data from plate reader for reduced NADP^+^ and oxidated NADPH
  - Background subtracted readings
  - Full western images, including ladder
    - Ponceau stains confirming protein transfer
    - ECL negative control from step 9-hr

#### Confirmatory analysis plan

Statistical Analysis of the Replication Data:

- Note: At the time of analysis, we will perform the Shapiro-Wilk test and generate a quantile-quantile plot to assess the normality of the data. We will also perform Levene’s test to assess homoscedasticity. If the data appears skewed, we will perform the appropriate transformation to proceed with the proposed statistical analysis. If this is not possible, we will perform the equivalent non-parametric Wilcoxon-Mann-Whitney test.
  - For oxidative activity assays:
    - Bonferroni corrected ANOVA followed by two-tailed Bonferroni corrected planned contrasts:
      - Vector vs. IDH2^WT^
      - Vector vs. IDH2^R172K^
  - For reductive activity assays
    - Bonferroni corrected ANOVA followed by two-tailed Bonferroni corrected planned contrasts:
      - Vector vs. IDH2^WT^
      - Vector vs. IDH2^R172K^
  - Western blot:
    - This is a quality control experiment and is not powered to detect a specific effect.
- Meta-analysis of original and replication attempt:
  - This replication attempt will perform the statistical analysis listed above, compute the effects sizes, compare them against the reported effect size in the original paper and use a meta-analytic approach to combine the original and replication effects, which will be presented as a forest plot.

#### Known differences from the original study

Although not performed by the original authors, actin was added as internal loading control for Western blots and will be added to the resulting data. Details of the Western blot protocol and possible stripping/sodium azide treatment were unspecified; information was added by the replicating lab. The details of the transfection specifics were unspecified and that information is provided by the replicating lab. Additionally, these experiments will be conducted in 6-well dishes, however, if total protein yield is not sufficient, the replicating lab will scale up to 25 cm dishes.

#### Provisions for quality control

All data obtained from the experiment - raw data, data analysis, control data, and quality control data - will be made publicly available, either in the published manuscript or as an open access dataset available on the Open Science Framework (https://osf.io/8l4ea/).

- STR profiling and mycoplasma testing results
- Sequencing reads and agarose gel images confirming vector identity and integrity
- Ponceau stains confirming protein transfer for Western Blot
- Confirmation of HRP inactivation prior to proceeding with the following antibodies.

### Protocol 2: Production of 2-HG from IDH2 WT and mutant transfected cells

In this protocol, the production of 2-HG from 293T cells transfected with vectors expressing IDH2^WT^ or IDH2^R172K^ is measured by gas chromatography-mass spectrometry (as seen in Figures 3A–C). The amount of 2-HG relative to glutamate is quantified, as seen in Figure 3D.

#### Sampling

- Experiment will be performed with at least three biological replicates for a final power of at least 80%. The original data are qualitative, thus to determine an appropriate number of replicates to initially perform, sample sizes based on a range of potential variance was determined.
  - See Power Calculations section for details.
- Experiment has three conditions:
  - Condition 1:293T cells expressing IDH2^WT^
  - Condition 2: 293T cells expressing IDH2^R172K^
  - Condition 3: 293T cells expressing empty pCDNA3 vector
- For each condition, lysates will be analyzed for 2-HG/glutamate levels

#### Materials and reagents

**Table:** 

Reagent	Type	Manufacturer	Catalog #	Comments
293T cells	Cells	ATCC	CRL-3216	Original source unspecified
Dulbecco’s modified Eagle’s medium (DMEM)	Media	Invitrogen	11965118	Original unspecified
Pen/Strep	Reagent	Fisher	15140-122	Original unspecified
FBS	Reagent	Hyclone	SH30071.03	Replaces FBS from CellGro
pcDNA-IDH2^WT^	Plasmid	Generated in Protocol 1		
pcDNA-IDH2^R172K^	Plasmid	Generated in Protocol 1		
Lipofectamine 2000	Reagent	Invitrogen	11668027	
Methanol	Reagent	Fisher	A452SK-4	Original unspecified
Refrigerated centrifuge	Equipment	Labnet International, Inc	PrismR	Original unspecified
Nitrogen gas	Reagent	Generated in lab		Original unspecified
AG-1 X8 100-200 anion exchange column	Reagent	Bio-Rad	731-6211	Poly-Prep Columns, AG 1-X8, chloride form
HCl	Reagent	Fisher	SA56-1	Original unspecified
N-methyl-N-tert- butyldimethylsily trifluoroacetamide (MTBSTFA; Regis)	Reagent	Regis	1-270243-200	
Gas Chromatograph with an HP-5MS capillary column and Mass selective detector	Equipment	Agilent 7890A with 7693 Autosampler		
Cold trap concentrator	Equiptment	Labconco Centrivap		
R(-)-2-HG	Reagent	Sigma-Aldrich	H8378-100MG	Original unspecified

#### Procedure

##### Notes

- 293T cells grown in DMEM supplemented with 10% FBS at 37°C in 5% CO_2_.
- All cells will be sent for STR profiling and mycoplasma testing

1. Seed 0.25–1 x 10^6^ 293T cells per well of a 6-well plate in growth medium without antibiotics.
  - Grow overnight.
  - Confirm cells at 70–80% confluence by light microscopy at time of transfection.
2. Transfect 293T cells with pCDNA3, pCDNA3-IDH2^WT^, or pCDNA3-IDH2^R172K^ with Lipofectamine 2000 according to manufacturer instructions.
  - Transfect 1 µg of plasmid DNA per well in 6-well plate at 70–80% confluence.
  - Generate duplicate plates for each transfection:
    - Harvest one plate at 24 hr.
    - Harvest one plate at 48 hr.
3. 24 hr later, replace with fresh media with 1x pen/strep
4. 24 or 48 hr later, gently remove medium from proliferating cells.
  - Note: from this point on this protocol contains information as described in (Bennett et al., 2008).
5. Rapidly quench cells with 1–2 ml per well of -80°C methanol.
  - Chill cells to -80°C and incubate at -80°C for 15 min.
6. Scrape cells off the dish and transfer the cell suspension to a 15 ml conical tube.
  - Centrifuge for 5 min at 2000x*g* at 4°C to pellet cellular debris.
  - Transfer supernatant to a fresh 15 ml tube.
7. Resuspend the pellet in 500 µl of -80°C 80% methanol in water by vortexing.
  - Incubate at 4°C for 15 min.
  - Centrifuge for 5 min at 2000x*g* at 4°C.
  - Combine supernatant with supernatant from Step 6b.
  - Repeat step 7 for a third round of extraction and combine all supernatants.
8. Evaporate to dryness using a cold trap concentrator.
9. Elute through an AG-1 X8 100–200 anion exchange resin according to the manufacturer’s instructions.
  - Wash with five column volumes of wash buffer.
  - Elute in 3N HCl.
10. Evaporate to dryness using cold trap concentrator
11. Redissolve sample in MSTFA + FAME.
  - Prepare 40 mg/mL Methoxyamine hydrochloride (MeOX) solution in pyridine.
    - Weigh out methoxyamine hydrochloride in 1.5 ml Eppendorf tube on balance and add appropriate amount of pyridine.
  - Vortex MeOX solution and sonicate at 60°C for 15 min to dissolve.
  - Add 10 µl of 40 mg/ml MeOX solution to each dried sample.
  - Shake at maximum speed at 60˚C for 1 hr.
  - To 1 ml of MSTFA, add 10 µl of FAME marker.
    - Vortex for 10 s.
  - Add 91 µl of MSTFA + FAME mixture to each sample and standard. Cap immediately.
    - Shake at maximum speed at 37°C.
  - Transfer contents to glass vials with micro-inserts and cap immediately.
    - Submit to GCTOF MS analysis.
12. Inject samples into GC-MS.
  - Operate the detector in spitless mode using electron impact ionization.
    - Ionizing voltage: -70 eV
    - Electron multiplier: 1060 V
  - GC temperature ramp:
    - Hold at 100°C for 3 min.
    - Ramp to 230°C at 4°C/min.
    - Hold for 4 min.
    - Ramp to 300°C.
    - Hold for 5 min.
  - Record mass range of 50–500 amu and record 2.71 scans/s.
13. Repeat steps 1–12 independently three additional times.

#### Deliverables

- Data to be collected:
  - 24 hr samples:
    - GC traces for all samples run
      - Close-up of the time range showing metabolite abundance for aspartate, glutamate, and 2-HG for cells transfected with IDH2^WT^ (Figure 3A) and cells transfected with IDH2^R172K^ (Figure 3B).
        - Mass spectrum confirmation of metabolite identity as 2-HG.
  - 48 hr run
    - GC traces for all samples run
      Close-up of the time range showing metabolite abundance for aspartate, glutamate, and 2-HG for cells transfected with IDH2^WT^ (Figure 3A) and cells transfected with IDH2^R172K^ (Figure 3B).
    - Quantification of the relative intensity of the 2-HG signal to the glutamate signal, graphed as seen in Figure 3D.

#### Confirmatory analysis plan

- Statistical Analysis of the Replication Data:
- Note: At the time of analysis, we will perform the Shapiro-Wilk test and generate a quantile-quantile plot to assess the normality of the data. We will also perform Levene’s test to assess homoscedasticity. If the data appears skewed, we will perform the appropriate transformation to proceed with the proposed statistical analysis. If this is not possible, we will perform the equivalent non-parametric test.
  - Two-way ANOVA performed on 2-HG/glutamate ratios followed by Fisher’s LSD for the following comparisons:
      - Vector vs. IDH2^WT^
      - IDH2^WT^ vs. IDH2^R172K^
    o Analyses will be performed on both 24 and 48 hr runs.
- Meta-analysis of original and replication attempt:
  - The replication data will be presented as a mean with 95% confidence intervals and will include the original data point, calculated directly from the graph, as a single point on the same plot for comparison.

#### Known differences from the original study

- The GC-MS sample preparation protocol was modified by the replicating lab including a shaking incubation step at 11f. However, this protocol was taken from Bennett et al. which the authors reference in the original manuscript.

#### Provisions for quality control

All data obtained from the experiment - raw data, data analysis, control data and quality control data - will be made publicly available, either in the published manuscript or as an open access dataset available on the Open Science Framework (https://osf.io/8l4ea/).

- STR profiling and mycoplasma testing results.
- Mass spectrum of the metabolite peak for derivatized 2HG to confirm identity.

### Protocol 3: Assessing the correlation of IDH status with 2-HG levels in samples from patients with AML

In this protocol, samples from patients with acute myeloid leukemia (AML) are examined for their IDH mutational status and their level of 2-HG, as seen in Figure 5.

#### Sampling

- This experiment will use four samples per group for a final power of at least 80%.
  - See Power Calculations section for details.
- This experiment has three genetically distinct groups:
  - AML patients with no *IDH* mutations
  - AML patients with mutant *IDH1*
  - AML patients with mutant *IDH2,* including both R172K and R140Q mutants
- All samples will come from Roswell Park Cancer Institute and are ficoll separated in media with 10% DMSO and prescreened for *IDH* genotypic status.
- Each patient sample will be assessed for their ratio of 2-HG/glutamate.

#### Materials and reagents

**Table:** 

Reagent	Type	Manufacturer	Catalog #	Comments
Samples of peripheral blood, bone marrow, or pheresis from patients with karyotypically normal AML	Patient sample	NA	NA	Banked RPCI samples
DMSO	Reagent	Fisher	BP231-1	Original Unspecified
Methanol	Reagent	Fisher	A452SK-4	Original unspecified
Refrigerated centrifuge	Equipment	Labnet International, Inc	PrismR	Original unspecified
AG-1 X8 100-200 anion exchange column	Reagent	Bio-Rad	731-6211	Poly-Prep Columns, AG 1-X8, chloride form
HCl	Reagent	Fisher	SA56-1	Original unspecified
N-methyl-N-tert- butyldimethylsily trifluoroacetamide (MTBSTFA; Regis)	Reagent	Regis	1-270243-200	
Gas Chromatograph with an HP-5MS capillary column and Mass selective detector	Equipment	Agilent 7890A with 7693 Autosampler		
Cold trap concentrator	Equiptment	Labconco Centrivap		

#### Procedure

1. GC-MS analysis of 2-HG levels.
  - If using frozen cells, warm cells to 37˚C in a 37˚C water bath for 10 min
  - Centrifuge cells for 5 min at 1000x*g* to form a pellet
    - If necessary, transfer cells to a conical or microcentrifuge tube
  - Gently remove freezing medium from MNCs
  - Proceed with metabolite extraction and GC-MS analysis as detailed in protocol 2 Steps 5 through 12.
  - For each sample, divide the GC signal intensity of their 2-HG peak by the signal intensity of their glutamate peak and graph.

#### Deliverables

- Data to be collected:
  - Tabulated patient data (age, sex, *IDH* mutation status, 2-HG/glutamate ratio) (as seen in Table 1)
  - GC traces for all samples
  - Graph of 2-HG/glutamate ratio for samples by mutational status, as seen in Figure 5C.

#### Confirmatory analysis plan

- Statistical Analysis of the Replication Data:
- Note: The authors report WT IDH ratios were less than 1% which we are using as the constant for the comparisons below.
  - Bonferroni Correct one-sample t-test for 3 comparisons (alpha corrected for 2 test groups = 0.025)
    - Constant vs. *IDH1^mutant^*
    - Constant vs. *IDH2^mutant^*
    - Constant vs *IDH1/2^mutants^*
- Meta-analysis of original and replication attempt:
  - This replication attempt will perform the statistical analysis listed above, compute the effects sizes, compare them against the reported effect size in the original paper and use a meta-analytic approach to combine the original and replication effects, which will be presented as a forest plot.

#### Known differences from the original study

- The GC-MS sample preparation protocol was modified by the replicating lab including a shaking incubation step at 11f, protocol 2. However, this protocol was taken from Bennett et al. which the authors reference in the original manuscript.

#### Provisions for quality control

All data obtained from the experiment - raw data, data analysis, control data and quality control data - will be made publicly available, either in the published manuscript or as an open access dataset available on the Open Science Framework (https://osf.io/8l4ea/). This includes confirmation of the GCMS peaks and elution times as well as MS QC data.

### Power calculations

For details of power calculations, see spreadsheet and additional files at https://osf.io/9jkpg/

#### Protocol 1

Summary of original data estimated from graph reported in Figure 2A:

- SD was calculated using formula SD = SEM*(SQRT n=3).

**Table:** 

Sample	Time	Mean	SEM	SD
IDH2^WT^	0	0	0.0820	0.1421
	5	0.225	0.0820	0.1421
	10	0.45	0.1025	0.1776
	15	0.679	0.1538	0.2664
	20	0.917	0.1974	0.3419
	25	1.129	0.2512	0.4352
	30	1.342	0.3	0.5196
IDH2^R172K^	0	0	0.0820	0.1421
	5	0.038	0.0820	0.1421
	10	0.062	0.0820	0.1421
	15	0.062	0.0820	0.1421
	20	0.062	0.0820	0.1421
	25	0.1	0.0820	0.1421
	30	0.096	0.0820	0.1421
Vector	0	0	0.0564	0.0977
	5	0.021	0.0564	0.0977
	10	0.021	0.0564	0.0977
	15	0.017	0.0564	0.0977
	20	0.017	0.0564	0.0977
	25	0.033	0.0564	0.0977
	30	0.021	0.0564	0.0977

Linear regression to determine slopes from estimate values.

Calculations performed with R software (version 3.2.2) (R Core Team, 2015)

**Table:** 

Sample	Mean slope	SD	N
IDH2^WT^	0.01	0.090	3
IDH2^R172K^	0.06	0.140	3
Vector	0.67	0.280	3

Summary of original data estimated from graph reported in Figure 2B:

- SD was calculated using formula SD = SEM*(SQRT(n)), where n = 3.

**Table:** 

Sample	Time	Original_Value_Mean	SEM	SD
IDH2^WT^	0	0	0.0039	0.0067
	17	-0.003	0.0060	0.0105
	33	-0.004	0.0073	0.0126
	50	-0.005	0.0102	0.0177
	71	-0.006	0.0104	0.0181
	90	-0.008	0.0114	0.0198
	112	-0.009	0.0117	0.0202
	131	-0.01	0.0075	0.0130
	171	-0.014	0.0121	0.0211
IDH2^R172K^	0	0	0.0039	0.0067
	17	-0.006	0.0039	0.0067
	33	-0.009	0.0065	0.0114
	50	-0.016	0.0085	0.0147
	71	-0.024	0.0080	0.0139
	90	-0.028	0.0087	0.0152
	112	-0.036	0.0095	0.0164
	131	-0.043	0.0104	0.0181
	171	-0.055	0.0095	0.0164
Vector	0	0	0.0026	0.0046
	17	0.001	0.0026	0.0046
	33	0	0.0026	0.0046
	50	0	0.0026	0.0046
	71	0	0.0026	0.0046
	90	0	0.0026	0.0046
	112	-0.002	0.0026	0.0046
	131	-0.002	0.0026	0.0046
	171	-0.003	0.0026	0.0046

Linear regression to determine slopes from estimates values.

Calculations performed with R software (version 3.2.2) (R Core Team, 2015)

**Table:** 

Sample	Mean slope	SD	N
IDH2^WT^	-0.0006	0.005	3
IDH2^R172K^	-0.0241	0.013	3
Vector	-0.0065	0.016	3

#### Test family

- One-way ANOVA: Fixed effects, omnibus, one-way: Bonferroni correction: alpha error = 0.025.

#### Power calculations

- Power calculations were performed using G*Power, version 3.1.7 (Faul et al., 2007).
- ANOVA F test statistic and partial η^2^performed with R software, version 3.2.2 (R Core Team, 2015).

**Table:** 

Groups	F test statistic	Partial η^2^	Effect size *f*	A priori power	Total sample size
Slopes of NADPH production from IDH2^WT^, IDH2^R172^, or Vector (Figure 2A)	F(2,6) = 10.8	0.7826	1.897636	99.99%^1^	21^1^ (3 groups)
Slopes of NADP^+^ production from IDH2^WT^, IDH2^R172^, or Vector (Figure 2B)	F(2,6) = 3.02	0.5023	1.0048	94.13%^1^	21^1^ (3 groups)

^1^ 7 samples per group will be used based on the planned comparisons making the power at least 80%.

#### Test family

- 2 tailed *t* test, Wilcoxon-Mann-Whitney test, Bonferroni’s correction: alpha error = 0.0125

Power Calculations performed with G*Power software, version 3.1.7 (Faul et al., 2007).

##### Figure 2A (NADPH production) values

**Table:** 

Group 1	Group 2	Effect size *d*	A priori power	Group 1 sample size	Group 2 sample size
Vector	IDH2^WT^	3.05134	98.8%^1^	7^1^	7^1^
Vector	IDH2^R172K^	2.12463^2^	80.0%^2^	7	7

^1^ 7 samples per group will be used based on the Vector vs IDH2^R172K^ NADP^+^ planned comparison making the power 98.8%.

^2^ A sensitivity calculation was performed since the original data showed a non-significant effect. This is the effect size that can be detected with 80% power and the indicated sample size. The original effect size reported was 0.49386.

##### Figure 2B (NADP^+^ production) values

**Table:** 

Group 1	Group 2	Effect size *d*	A priori power	Group 1 sample size	Group 2 sample size
Vector	IDH2^WT^	2.12463^1^	80.0%^1^	7	7
Vector	IDH2^R172K^	2.21471	89.3%	7	7

^1^ A sensitivity calculation was performed since the original data showed a non-significant effect. This is the effect size that can be detected with 80% power and the indicated sample size. The original effect size reported was 0.47369.

#### Test family

- Due to the large variance, these parametric tests are only used for comparison purposes. To ensure an adequate sample size is used, the number is based on the non-parametric tests listed above.
- 2 tailed *t* test, difference between two independent means, Bonferroni’s correction: alpha error = 0.0125

Power Calculations performed with G*Power software, version 3.1.7 (Faul et al., 2007).

##### Figure 2A (NADPH production) values

**Table:** 

Group 1	Group 2	Effect size *d*	A priori power	Group 1 sample size	Group 2 sample size
Vector	IDH2^WT^	3.05134	99.2%^1^	7^1^	7^1^
Vector	IDH2^R172K^	2.03^2^	80.0%^2^	7	7

^1^Seven samples per group will be used based on the Vector vs IDH2^R172K^ NADP^+^ planned comparison making the power 98.8%.

^2^ A sensitivity calculation was performed since the original data showed a non-significant effect. This is the effect size that can be detected with 80% power and the indicated sample size. The original effect size reported was 0.33972.

##### Figure 2B (NADP^+^ production) values

**Table:** 

Group 1	Group 2	Effect size *d*	A priori power	Group 1 sample size	Group 2 sample size
Vector	IDH2^WT^	2.05829^1^	80.0%^1^	7	7
Vector	IDH2^R172K^	2.03	90.4%	7	7

^1^ A sensitivity calculation was performed since the original data showed a non-significant effect. This is the effect size that can be detected with 80% power and the indicated sample size. The original effect size reported was 0.51213.

### Protocol 2: Figure 3D

Summary of original data

- Note: data estimated from published graphs

**Table:** 

Sample	Mean intracellular 2-HG/glutamate	Assumed N
Vector	0.0105	3
IDH2^WT^	0.0102	3
IDH2^R172K^	1.2	3

#### Test family

- One way ANOVA followed by Bonferroni corrected planed comparisons:
  - Power calculations:
    - Vector vs. IDH2^R172K^
    - IDH2^WT^ vs. IDH2^R172K^
  - Sensitivity Calculations
    - Vector vs. IDH2^WT^

#### Power calculations

- Power calculations were performed using GraphPad PRISM v6 and G*Power (version 3.1.7) (Faul et al., 2007)
- Because the data did not display variance, we have performed power calculations with a range of variances and an assumed N of 3 per group.
- 2% variance

**Table:** 

ANOVA; α=0.05				
F(2,6)	Partial eta2	Effect size f	Power	Total N
7370	0.999593	49.55807	>99.99%	6*
Power calculations; α=0.05				
Group 1	Group 2	Effect size d	Power	N/group
Vector	IDH2^WT^	70.10710478	>99.99%	2*
IDH2^WT^	IDH2^R172K^	70.08927663	>99.99%	2*
Sensitivity Calculations; α=0.05, powered to 80%				
Group 1	Group 2	Effect size d	Detectable d	N/group
Vector	IDH2^R172K^	1.449123183	0.2774844	3

*With a minimum of 3 per group (9 total), achieved power is >99.99%.

- 15% variance

**Table:** 

ANOVA; α=0.05				
F(2,6)	Partial eta2	Effect size f	Power	Total N
131	0.977612	6.608085	99.99%	6*
Power calculations; α=0.05				
Group 1	Group 2	Effect size d	Power	N/group
Vector	IDH2^WT^	9.347613971	98.65%	2*
IDH2^WT^	IDH2^R172K^	9.345236884	98.65%	2*
Sensitivity Calculations; α=0.05, powered to 80%				
Group 1	Group 2	Effect size d	Detectable d	N/group
Vector	IDH2^R172K^	0.193216424	0.0539826	3

*With a minimum of 3 per group (9 total), achieved power is >99.99%.

- 28% variance

**Table:** 

ANOVA; α=0.05				
F(2,6)	Partial eta2	Effect size f	Power	Total N
37.60	0.926108	3.540235	98.61%	6*
Power calculations; α=0.05				
Group 1	Group 2	Effect size d	Power	N/group
Vector	IDH2^WT^	5.007650342	99.28%	3
IDH2^WT^	IDH2^R172K^	5.006376902	99.28%	3
Sensitivity Calculations; α=0.05, powered to 80%				
Group 1	Group 2	Effect size d	Detectable d	N/group
Vector	IDH2^R172K^	0.103508799	0.0511419	3

*With a minimum of 3 per group (9 total), achieved power is 99.99%.

- 40% variance

**Table:** 

ANOVA; α=0.05				
F(2,6)	Partial eta2	Effect size f	Power	Total N
18.43	0.860009	2.478571	85.73%	6*
Power calculations; α=0.05				
Group 1	Group 2	Effect size d	Power	N/group
Vector	IDH2^WT^	3.505355239	88.73%	3
IDH2^WT^	IDH2R172K	4.205285771	96.37%	3
Sensitivity Calculations; α=0.05, powered to 80%				
Group 1	Group 2	Effect size d	Detectable d	N/group
Vector	IDH2^R172K^	0.072456159	0.0505594	3

*With a minimum of 3 per group (9 total), achieved power is 99.92%.

- In order to produce quantitative replication data, we will run the experiment three times. We will determine the standard deviation across the biological replicates and combine this with the reported value from the original study to simulate the original effect size. We will use this simulated effect size to determine the number of replicates necessary to reach a power of at least 80%. We will then perform additional replicates, if required, to ensure that the experiment has more than 80% power to detect the original effect.
- Note: Simulation analysis was also conducted using randomly generated values based on the SD and variance desired. These data are comparable to what is seen above when using a parametric model approach. Also there may be a need to appropriately transform these data based on the scale of Figure 3D, and we have assumed that this is one representative sample and not averages of all the data showing no variance. This simulation will be loaded to the OSF (https://osf.io/8l4ea/).

### Protocol 3: Figure 5C

#### Summary of original data

- Note: data estimated from published graphs and log transformed. Data includes IDH^WT^ (no mutations in *IDH1* or *IDH2*), IDH1^R132C/G^, IDH2^Mutant^ (IDH2^R172K^ and IDH2^R140Q^)

**Table:** 

Sample	2HG/glutamate	log(2HG/glut)
IDH^WT^(Constant)	0.01	-4.605
IDH1^Mutant^	0.600	-0.511
IDH1^Mutant^	1.200	0.182
IDH1^Mutant^	1.600	0.470
IDH1^Mutant^	1.800	0.588
IDH1^Mutant^	3.000	1.099
IDH1^Mutant^	0.600	-0.511
IDH2^Mutant^	0.140	-1.966
IDH2^Mutant^	0.160	-1.832
IDH2^Mutant^	0.290	-1.237
IDH2^Mutant^	0.300	-1.204
IDH2^Mutant^	0.310	-1.171
IDH2^Mutant^	0.470	-0.755
IDH2^Mutant^	0.590	-0.528
IDH2^Mutant^	0.310	-1.171

#### Test family

- One sample t-test comparing Constant and mutant *IDH* groups:
  - Constant vs. *IDH1^R132C/G^*
  - Constant vs. *IDH2^mutant^* (grouped)
  - Constant vs. *IDH1/2^mutant ^*(grouped)

#### Power calculations

Power calculations were performed using R software version 3.2.2 and G*Power (version 3.1.7) (Faul et al., 2007). Bonferroni corrected one-sample *t*-tests compared to. 01 (threshed as reported by original authors).

**Table:** 

Constant	Group	Effect size *d*	A priori power	Group sample size
0.01	IDH1^R132C/G^	8.404	99.99%	4
0.01	IDH2^Mutant^	6.746	99.99%	4
0.01	IDH1/2^Mutant^	4.361	99.99%	4

- Because of the inherent complications that can occur when using primary patient cell lines, we have adjusted our sample size to four samples/group even though we achieve >90% power when using three samples/group.
